# Synaptoprotection in Perinatal Asphyxia: An Experimental Approach

**DOI:** 10.3389/fnsyn.2020.00035

**Published:** 2020-09-23

**Authors:** María Inés Herrera, Tamara Kobiec, Rodolfo Kölliker-Frers, Matilde Otero-Losada, Francisco Capani

**Affiliations:** ^1^Centro de Investigaciones en Psicología y Psicopedagogía (CIPP), Facultad de Psicología y Psicopedagogía, Universidad Católica Argentina (UCA), Buenos Aires, Argentina; ^2^Centro de Altos Estudios en Ciencias Humanas y de la Salud (CAECIHS), Universidad Abierta Interamericana, Buenos Aires, Argentina; ^3^Consejo Nacional de Investigaciones Científicas y Técnicas (CONICET), Buenos Aires, Argentina; ^4^Facultad de Psicología y Psicopedagogía, Universidad Católica Argentina, Buenos Aires, Argentina; ^5^Departamento de Biología, Universidad Argentina John F. Kennedy, Buenos Aires, Argentina; ^6^Facultad de Ciencias de la Salud, Universidad Autónoma de Chile, Santiago de Chile, Chile

**Keywords:** perinatal asphyxia, animal model, synaptopathy, neuroprotective strategies, synaptoprotection

## Abstract

Perinatal asphyxia (PA) is an obstetric complication occurring when the oxygen supply to the newborn is temporally interrupted. This health problem is associated with high morbimortality in term and preterm neonates. It severely affects the brain structure and function, involving cortical, hippocampal, and striatal loss of neurons. Nearly 25% of PA survivor newborns develop several neurodevelopmental disabilities. Behavioral alterations, as well as the morphological and biochemical pathways involved in PA pathophysiology, have been studied using an animal model that resembles intrauterine asphyxia. Experimental evidence shows that PA induces synaptic derangement. Then, synaptic dysfunction embodies a putative target for neuroprotective strategies. Over the last years, therapeutic hypothermia (TH), the only treatment available, has shown positive results in the clinic. Several pharmacological agents are being tested in experimental or clinical trial studies to prevent synaptopathy. Preservation of the synaptic structure and function, i.e., “synaptoprotection,” makes up a promising challenge for reducing incidental neurodevelopmental disorders associated with PA. Accordingly, here, we summarize and review the findings obtained from the referred experimental model and propose a renewed overview in the field.

## Introduction

**Figure 1:**
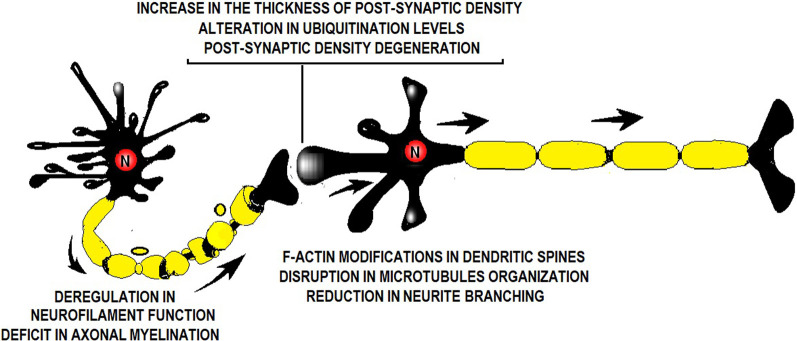
Illustration showing a synapse and the factors altered by perinatal asphyxia (PA). PA alters neurofilament function, axonal myelination, postsynaptic density thickness, ubiquitination level, dendritic spine ultrastructure, microtubule organization, and neurite branching.

**Figure 2:**
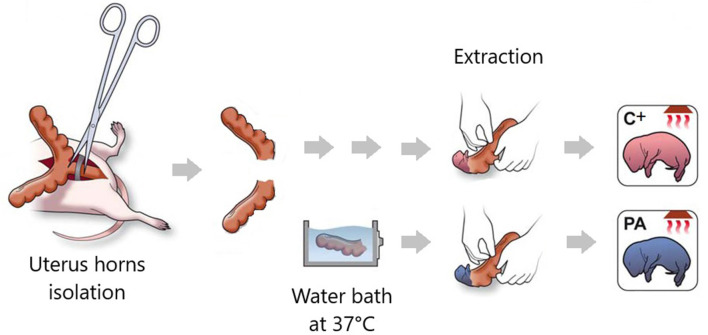
Illustration summarizing the main procedures in the Swedish experimental model of PA. Pregnant rats that have delivered no more than two pups are hysterectomized. Uterus horns are isolated. One of them is opened and the pups are removed (pups born by cesarean section, C+ group). The other uterus horn is immersed in a water bath at 37°C for 14–19 min (pups born by cesarean section plus PA group). Schematic illustration adapted from Galeano et al. (2011).

Perinatal asphyxia (PA) is the obstetric complication resulting from a transient interruption of the oxygen supply to the newborn (Adcock and Papile, 2008). It remains a major determinant of mortality and morbidity in children despite neonatal care advances (Golubnitschaja et al., 2011). While research has, by large, focused on neuronal death soon after the asphyctic insult, the last decade has geared toward addressing the effects of PA on the synapses as well. Scientific evidence suggests that PA induces a “synaptopathy,” associated with alterations in postnatal neurogenesis and plasticity (Scheepens et al., 2003b; Tapia-Bustos et al., 2017), reduction in neurite length and branching (Klawitter et al., 2007; Rojas-Mancilla et al., 2013), changes in the level of neurotrophic factors and synaptic markers (Scheepens et al., 2003a,b; Weitzdörfer et al., 2008; Rojas-Mancilla et al., 2013), myelination deficit (Kohlhauser et al., 2000), protein misfolding, aggregation and ubiquitination (Capani et al., 2009; Grimaldi et al., 2012; Saraceno et al., 2012a,b, 2016; Muñiz et al., 2014), and presynaptic (Van de Berg et al., 2000) and postsynaptic density thickening (Capani et al., 2009; Grimaldi et al., 2012; Saraceno et al., 2012a,b, 2016; Muñiz et al., 2014; Figure 1). These synaptic alterations have been studied over the last 28 years (Barkhuizen et al., 2017) using a Swedish experimental PA model developed at the Karolinska Institutet. Pregnant rats are anesthetized and hysterectomized on the last gestation day, performing a delayed cesarean section that resembles delivery labor. Asphyxia is induced at postnatal day 0 by immersing fetus-containing uterus horns in a 37°C water bath for 14–19 min. Next, the pups are taken out, assisted to breathe on their own, and given to surrogate mothers in small experimental groups (Bjelke et al., 1991; Figure 2).

Rodent brain development at postnatal days 0–3 resembles gestation weeks 23–32 in humans, so this model applies to premature infants. During this time, the blood-brain barrier settles, and pre-oligodendrocytes are plentiful. In rat postnatal days 7–10, gliogenesis peaks, dendritic and axonal density increases, and immature oligodendrocytes prevail. These are developmental milestones observed at human gestation weeks 36–40. Around postnatal days 20–21, the myelination rate and synaptic density peak close to 50% of the adult levels, resembling a 2- to 3-year-old infant. In humans, from 4 to 11 years of age, the prefrontal cortex neural networks approach structural maturation, and gray matter reaches its largest volume. These benchmarks develop from postnatal days 25–35 in the rodent brain. Synaptic density decreases between postnatal days 35 and 49, reaching adult levels, while myelination and white matter continue maturing, as in human teens. Finally, from postnatal day 60 on, synaptic density and neurotransmitters meet adult levels, myelination proceeds, and gray matter declines, as in adult human life (Semple et al., 2013).

The best-known PA rat experimental models are Rice–Vannucci’s (Vannucci and Vannucci, 2005) and the Swedish’s (Bjelke et al., 1991). Both have strengths and disadvantages, depending on the aims of the research. Instead of immersing fetus-containing uterus horns in a 37°C water bath for 14–19 min, the Rice–Vannucci model induces hypoxic–ischemic injury by carotid artery ligation followed by an exposition to hypoxic air (8% oxygen) at 37°C for 3.5 h. It is useful for studying hypoxic–ischemic insult in term and early-term human neonates, for it is conducted in 7-day-old pups. Besides, in contrast with the Swedish model, brain damage is unilateral unless bilateral ligation is performed, allowing between-brain hemisphere comparison and a clear injury boundary (Rumajogee et al., 2016). Conversely, the experimental model developed by Bjelke and collaborators induces a global injury, diffuse, more like human PA, and its pathophysiology. The translational value stands out (Barkhuizen et al., 2017) for inducing asphyxia at the time of delivery, mimicking the prevalent delayed labor and umbilical cord coiling clinical features (Bjelke et al., 1991; Herrera-Marschitz et al., 2014). Besides its well-known suitability in assessing the behavioral, morphological, and biochemical effects of PA (Hoeger et al., 2006), the Swedish model allows easy complementation with *in vitro* studies. Also, this experimental approach has been used to study short- and long-term effects after PA. It shows high between-laboratory reproducibility (Barkhuizen et al., 2017) and has contributed to shedding light on the neuroprotective potential of several agents in preventing PA-induced synaptopathy (Herrera-Marschitz et al., 2011). Our work revises these findings using the Swedish experimental model and discusses their scientific relevance to the current paradigm of synaptoprotection, stressing on the need of preserving synaptic structure and function beyond neuronal survival (Coleman et al., 2004; Yang and Cynader, 2014; Li et al., 2016; Ryskamp et al., 2019).

## Experimental Findings Supporting PA-Induced Synaptic Alterations

In 1991, Bjelke et al. (1991) reported the impact of the hypoxic insult on dopamine neurotransmission circuitries while setting the experimental model of global asphyxia. Dopaminergic synapse dysfunction was concomitant with a time-dependent loss of pyramidal neurons in the hippocampal CA1 and CA3 regions in rats of 3 weeks of age. Original findings regarding the hippocampal architecture and the dopamine system were confirmed and extended at the Herrera-Marschitz Laboratory. This Chilean scientist had worked with Bjelke at the Karolinska Institutet, studying PA-induced alterations in dopamine circuitries. According to their findings, the next days after the insult, the number of dopaminergic neurons, branching, and the neurite length decreased largely *in vitro* (Klawitter et al., 2007). Consistently, *in vivo* studies revealed that, at postnatal day 30 (P30), PA produced a decrease in neurite length and branching and a reduction in the expression of synaptophysin and postsynaptic density protein 95 (PSD95), pre- and post-synaptic markers, respectively (Rojas-Mancilla et al., 2013). Recent findings on the first 3 days after PA suggested that the hypoxic insult might hinder the neurogenic process mediated by dopamine receptors, disrupting postnatal plasticity (Tapia-Bustos et al., 2017).

Furthermore, synaptic alterations were found in the hippocampus. According to expected changes in the protein levels during normal hippocampal development (Weitzdörfer et al., 2006), synapsin IIb showed a considerable increase in the first week after PA (Weitzdörfer et al., 2008). This increment was considered as a result of PA-induced compensatory synaptogenesis (Weitzdörfer et al., 2008). Contemporary studies suggest that compensatory mechanisms, which involve mitogenic and apoptotic proteins, attempt to counterbalance neuronal loss (Morales et al., 2008). Developmental changes during the first 28 days after PA also embrace a delayed increase in hippocampal cellular proliferation, likely related to alterations in neurotrophic factors (Scheepens et al., 2003b). In contraposition to the response observed in adult injury, PA was found to provoke opposing modifications on neurotrophic factors in the immature brain, as could be inferred from the nerve growth factor (NGF) and brain-derived neurotrophic factor (BDNF) contents (Scheepens et al., 2003a).

In our laboratory, we committed to studying protein array in postsynaptic densities (PSDs) following PA. In rats of 6 months of age, we found a long-term increase in striatal PSDs, associated with high ubiquitination level (Capani et al., 2009). The hippocampus underwent similar changes 4 months after PA (Saraceno et al., 2012b). Further findings showed that the early increase in striatal (Grimaldi et al., 2012) and hippocampal PSDs (Saraceno et al., 2016) observed 1 month after PA might precede the long-term changes. These studies from our laboratory, developed between 2009 and 2016, showed protein ubiquitination as a likely early biomarker of neurodegeneration, 1 month after the insult. This aberrant morphological mechanism might underlie PA-induced synaptic dysfunction (Capani et al., 2009; Grimaldi et al., 2012). The so-called synaptopathy concurs with irregular alterations in F-actin levels, as found in dendritic spines 4 months after PA (Saraceno et al., 2012a,b, 2016; Muñiz et al., 2014). This prior attempt to rescue neural tissue *via* synaptogenesis might not work according to the behavioral results. In short, over-plasticity might spoil the proper establishment of neural wiring along neurodevelopment (Saraceno et al., 2016). Our findings suggest that ultrastructural changes depend on the duration and severity of the injury. In agreement, Cebral and Loidl (2011) confirmed that striatal and hippocampal thickening in PSDs at postnatal day 45 was proportional to the perinatal insult span and severity. Alterations in the presynaptic locus also represent an interesting issue in the understanding of synaptic alterations after brain injury. Aged rats subjected to PA exhibited a high density of cortical and striatal presynaptic buttons 22 months after PA. Paradoxically, this attempt to counterbalance neuronal loss was associated with an exacerbation of the cognitive decline expected for their age (Van de Berg et al., 2000).

Recent evidence from our laboratory revealed that PA resulted in a short-term reduction in microtubule-associated protein 2 (MAP-2) immunostaining in the CA1 hippocampal area (Herrera et al., 2018) and the corpus striatum (Udovin et al., 2020), as was found at P30. Protein expression analysis by Western blot confirmed this dendritic alteration. Moreover, immunohistochemistry and Western blot showed deregulation of axonal phosphorylated high- and medium-molecular-weight neurofilament chains (pNF-H/M) in the hippocampus (Herrera et al., 2018) and the corpus striatum (Udovin et al., 2020) 1 month after PA. The changes in the respective dendritic and axonal markers were correlated with decreased vertical exploration and alterations in anxiety levels, inferred from prototypical behaviors in animals like rearing and grooming (Herrera et al., 2018).

There is a massive loss of GABAergic projection neurons and interneurons in rats of 3 and 6 weeks of age (Van de Berg et al., 2003) and 7 and 21 days old (Dell’Anna et al., 1996) in the corpus striatum and hippocampus following PA. This can lead to neuronal network disorganization and disruption, reducing the number of neurons and synaptic contacts later in life (Van de Berg et al., 2003). Oligodendrocyte precursor cells, observed in large numbers at the time of PA induction in this model, are also vulnerable, dying due to exposure to high intercellular glutamate levels (excitotoxicity). Oligodendrocyte precursors proliferate and differentiate into mature myelin-forming oligodendrocytes. Perinatal asphyxia affects this normal development, leading to impaired myelination. Long-term myelination deficits were found in the hippocampus and cerebellum because of PA induction, along with neuronal loss, according to the decreased neurofilament immunoreactivity 3 months after the injury (Kohlhauser et al., 2000). Myelination deficits affect nerve conduction velocity and disrupt synaptic transmission. Then, white matter alterations pose a relevant dysfunction regarding PA-induced synaptopathy in the developing brain (Herrera et al., 2017) and is one of the most common neuropathologies in premature infants (Barateiro et al., 2016; Singh et al., 2018). More details on the experimental findings of PA-induced synaptic alterations may be consulted in a related review of ours (Herrera et al., 2017).

## Neuroprotective Strategies in Experimental PA

Endogenous compensatory mechanisms observed after PA (Van de Berg et al., 2000; Morales et al., 2008; Weitzdörfer et al., 2008; Saraceno et al., 2016) may not be successful (Van de Berg et al., 2000; Saraceno et al., 2016). Therefore, several neuroprotective treatments have been studied using the Swedish experimental model (Herrera-Marschitz et al., 2011). Neuroprotective strategies are designed for early intervention in the clinical setting to mitigate secondary injury processes by seizing the therapeutic window within the first life-hours (Blanco et al., 2011).

### Therapeutic Hypothermia

Scientific findings using the referred experimental model have contributed to confirming the efficacy of cooling after the hypoxic insult. Therapeutic hypothermia (TH) is, so far, the only neuroprotective treatment for PA available and suitable in the clinical context. In the model of Bjelke et al. (1991), TH can be induced during PA (co-hypoxic TH) or after it (post-hypoxic TH). In the first case, meanwhile one of the uterine horns, still containing the fetuses, is placed in a 37°C water bath, the other horn is placed in a bath at 15°C, and then the experiment continues as explained before. In the post-hypoxia TH procedure, immediately after PA, the animals are placed on a clean plastic pad filled with crushed ice and cooled (whole body) from 37°C to 32°C (monitoring rectal temperature) under anesthesia. When the desired temperature is reached, the animals are removed from the cooling pad and placed on a heating pad to allow slow re-warming. Decreasing the temperature of the experimental environment prevented synaptic changes (Capani et al., 1997, 2009; Cebral et al., 2006; Hoeger et al., 2006; Cebral and Loidl, 2011; Muñiz et al., 2014). Adult rats that had been treated with TH at 15°C for 20 or 100 min showed a significant decrease in PA-induced dense electronic bodies surrounding the synaptic vesicles and dendrites in the corpus striatum and the neocortex. At 6 months of age, TH-treated asphyctic rats were comparable to control rats, according to the light and electron microscopy results of immunolabeling experiments (Capani et al., 1997). Similar findings were reported in the same hypothermic conditions after studying the long-term ultrastructural changes in neurofilaments in the neostriatum of asphyctic rats. Chronic cytoskeletal alterations observed in the neostriatal neurons of 6-month-old rats were prevented by TH (Cebral et al., 2006). Hypothermia, at 15°C for 20 min, also avoided the overproduction of ubiquitin–protein conjugates (ubi-proteins) induced by PA in rats of 6 months of age (Capani et al., 2009). Treatment in the same hypothermic conditions prevented PSD thickening induced by PA in the neostriatum and the hippocampus at postnatal day 45 (Cebral and Loidl, 2011). Later studies using 2-month-old rats showed that the long-term increase in PSDs derived from F-actin accumulation in the neostriatal dendritic spines could be blocked or mitigated by co-hypoxic (at 15°C for 20 min) or post-hypoxic (at 32°C for 20 min) hypothermia, respectively. Regardless of the timing, birth hypothermia prevented synaptic cytoskeletal disorganization (Muñiz et al., 2014). The neuroprotective role of TH showed inconsistent results depending on the respective level of analysis. Although cooling could improve motor and cognitive function, the brain morphology was anyway altered in rats of 3 months of age, in different hypothermic conditions (Hoeger et al., 2006). Since neuroprotection by hypothermia is partial, pharmacological agents have been studied in the last decades as potential coadjuvant therapy for neonates suffering from PA (Cilio and Ferriero, 2010; Table 1).

**Table 1:** Neuroprotective strategies in perinatal asphyxia (PA): summary of experimental findings.

Neuroprotective strategy	Time after PA	Brain area	Main findings	Reference
Therapeutic hypothermia (TH)	6 months	Striatum, neocortex	TH reduced morphological damage in synaptic vesicles and dendrites.	Capani et al. (1997)
	6 months	Neostriatum	TH prevented neuronal cytoskeletal alterations.	Cebral et al. (2006)
	3 months	Hippocampus, hypothalamus	Co-hypoxic hypothermia protected rats from the functional, but not the morphological, effects of PA.	Hoeger et al. (2006)
	6 months	Neostriatum	TH revealed protective effects against overproduction of ubi-protein conjugates.	Capani et al. (2009)
	45 days	Neostriatum, hippocampus	TH inhibited the increase in PSD thickness.	Cebral and Loidl (2011)
	2 months	Neostriatum	Co- and post-hypoxic hypothermia prevented actin change in dendritic spines and neuronal death.	Muñiz et al. (2014)
Nicotinamide administration	3 days	Substantia nigra, neostriatum, neocortex	Nicotinamide prevented the decrease in the number of nNOS+ cells and neurite length and prevented PA effect on TH+ neurite length	Klawitter et al. (2007)
SKF38393 and quinpirole administration	1–3 days	Hippocampus subventricular zone	Cell death was reduced by both SKF38393 and quinpirole treatment. Neurogenesis was increased by quinpirole in the hippocampus.	Tapia-Bustos et al. (2017)
17β-estradiol administration	4 months	Hippocampus	Estradiol reversed focal swelling and the fragmented appearance of MAP-2 immunoreactive dendrites, along with the decrease in MAP-2 immunoreactivity.	Saraceno et al. (2010)
	4 months	Hippocampus	Estradiol showed neuroprotective effects *via* the interaction between ERα and IGF-IR, with the subsequent downstream activation.	Saraceno et al. (2018)
Palmitoylethanolamide (PEA) administration	1 month	Hippocampus	PEA treatment attenuated hippocampal damage (decrease in dendritic MAP-2 levels and accumulation of axonal pNF-H/M) and its correlated behavioral alterations.	Herrera et al., (2018)
	1 month	Striatum	PEA reduced the decrease in MAP-2 and pNF-H/M reactive areas, reversed the decrease in MAP-2 expression, and prevented pNF-H/M depletion.	Udovin et al. (2020)

### Nicotinamide, SKF38393, and Quinpirole Administration

Regarding other neuroprotective strategies, nicotinamide is a promising potential agent for preventing synaptic neostriatal spoilage induced by energy deficits like the decrease in nNOS (neuronal nitric oxide synthase) and TH+ (tyrosine hydroxylase) neurite length on the first days after PA (Klawitter et al., 2007). Dopamine receptor agonists have been tested for neuroprotection in organotypic cultures from control and asphyxia-exposed rats. On the first three postnatal days, quinpirole and SKF38393, D2/D3, and D1/D5 agonists, respectively, inhibited PA-induced increase in cell death in the hippocampus and the subventricular zone (SVZ). Quinpirole also increased neurogenesis in the hippocampus, antagonized by sulpiride, another D2 receptor. This interesting finding suggests that dopamine might modulate hippocampal postnatal neurogenesis mainly *via* D2 receptors (Tapia-Bustos et al., 2017; Table 1).

### 17β-Estradiol Administration

The estrogenic compound 17β-estradiol has shown neuroprotective properties against the decrease in MAP-2 level, focal swelling, and fragmented appearance of MAP-2 immunoreactive dendrites in the CA1 area of the hippocampus 4 months after PA. Late administration of 250 μg/kg for 3 days reversed the hippocampal damage (Saraceno et al., 2010). Further studies explored the mechanism underlying the therapeutic effect of 17β-estradiol treatment in rats of 4 months of age. The signaling pathway includes a rise in estrogen receptor (ER)-α and insulin-like growth factor (IGF-1R) protein level, upregulation of phosphatidylinositol 3-kinase/Akt/glycogen synthase kinase, 3 beta/β-catenin, and an increase in the Bcl-2/Bax ratio in the hippocampus. These findings suggest that the interaction between ER-α and IGF-1R, and the subsequent downstream activation, represents the mechanism of action of 17β-estradiol treatment in PA (Saraceno et al., 2018; Table 1).

### Palmitoylethanolamide Administration

Ethanolamine and palmitic acid react, giving rise to palmitoylethanolamide (PEA), a lipid compound that has shown protective effects after PA. Treatment with PEA (10 mg/kg) within the first hour of life reduced dendritic MAP-2 level and led to the deregulation of pNF-H/M axonal level in the CA1 hippocampal area (Herrera et al., 2018) and the corpus striatum (Udovin et al., 2020) 1 month after PA (Table 1). The morphological and biochemical neuroprotective effects in CA1 were associated with the improved exploratory activity and anxiety mitigation at P30. This time point in rats is equivalent to 4–11 years of age in humans, the period of neurodevelopmental disabilities onset (Semple et al., 2013).

## Synaptoprotection in PA? Translational Overview and Future Directions

Experimental findings using the Swedish rodent model have confirmed that birth asphyxia induces a synaptopathy (Herrera et al., 2017). Several neuroprotective strategies have shown beneficial effects, each one improving some aspect of the synaptic complex (Table 1). The question is whether they may be considered “synaptoprotective” agents, attempting to preserve synaptic structure and function beyond neuronal integrity. In fact, synaptic dysfunction might take place long before neurodegeneration, requiring “synaptoprotection” at a critical time point before the onset of clinical symptoms. This theoretical perspective, traced in the context of Alzheimer’s disease (Coleman et al., 2004), offers an interesting viewpoint for the progressive neurological dysfunction originating early in life (Marriott et al., 2017) following PA. Synaptoprotective treatments in PA neonates should target short-term markers preceding chronic signs of neurodegeneration (Capani et al., 2009; Saraceno et al., 2012b). The focus should be on protein damage (Grimaldi et al., 2012) in F-actin (Saraceno et al., 2012a, 2016), MAP-2 and pNF-H/M (Herrera et al., 2018; Udovin et al., 2020), reductions in neurite length and branching, and decreases in synaptophysin and PSD95 levels (Rojas-Mancilla et al., 2013) found 1 month after PA. Earlier indicators of PA-induced synaptic disorganization, like the change in NGF and BDNF contents in the first weeks of life, are also relevant. Growth factors are involved in the control of postnatal plasticity during impaired neurodevelopment and recovery from neonatal injury (Scheepens et al., 2003a), two intertwined processes (Volpe, 2009) that result in developmental disabilities (Zigmond et al., 2014). Following up over the first weeks of life becomes imperative, focusing on weight gain, appearance, and integration of reflexes, which are functional parameters of synaptic coherence (Kiss et al., 2009). Further studies involving biochemical, morphological, and behavioral outcomes along neurodevelopment are necessary to explain the synaptoprotective role of therapeutic interventions. For this purpose, the Swedish experimental model offers a useful paradigm from a translational perspective (Barkhuizen et al., 2017) according to benchmarks of maturation and vulnerability to injury between rodents and humans (Semple et al., 2013).

## Author Contributions

MH and MO-L: conception and original idea. MH, TK, and RK-F: investigation. MH and TK: original drafting. MO-L: critical revision and editing. FC: funding acquisition. The authors are accountable for the accuracy and integrity of all aspects of the work.

## Conflict of Interest

The authors declare that the research was conducted in the absence of any commercial or financial relationships that could be construed as a potential conflict of interest.
